# The role of alternative polyadenylation in breast cancer

**DOI:** 10.3389/fgene.2024.1377275

**Published:** 2024-06-13

**Authors:** Ping Qiao, Caihong Zhang, Yingxu Shi, Hua Du

**Affiliations:** ^1^ Department of Laboratory, Affiliated Hospital of Inner Mongolia Medical University, Hohhot, China; ^2^ Department of Pathology, Affiliated Hospital of Inner Mongolia Medical University, Hohhot, China; ^3^ College of Basic Medicine, Inner Mongolia Medical University, Hohhot, China

**Keywords:** alternative polyadenylation, APA, precursor mRNA, post transcriptional regulation, breast cancer

## Abstract

Breast cancer (BC), as a highly prevalent malignant tumor worldwide, is still unclear in its pathogenesis and has poor therapeutic outcomes. Alternative polyadenylation (APA) is a post-transcriptional regulatory mechanism widely found in eukaryotes. Precursor mRNA (pre-mRNA) undergoes the APA process to generate multiple mRNA isoforms with different coding regions or 3′UTRs, thereby greatly increasing the diversity and complexity of the eukaryotic transcriptome and proteome. Studies have shown that APA is involved in the progression of various diseases, including cancer, and plays a crucial role. Therefore, clarifying the biological mechanisms of APA and its regulators in breast cancer will help to comprehensively understand the pathogenesis of breast cancer and provide new ideas for its prevention and treatment.

## 1 Introduction

Breast cancer is currently a highly prevalent malignant tumor worldwide and is the most common cause of cancer death in women (Cao et al., 2021). In recent years, the incidence of breast cancer has been increasing at a rate of 0.5% per year (Siegel et al., 2021). Current treatments for BC mainly include surgery, radiotherapy, and endocrine therapy. However, because its specific pathogenesis is still unclear, the treatment effect is poor in clinical practice. Therefore, there is an urgent need to elucidate the molecular mechanisms of breast cancer pathogenesis to facilitate the search for more effective therapeutic methods and targets.

Uncontrolled cell proliferation characterizes cancer and is usually the result of aberrant gene expression. Studies have shown that post-transcriptional dysregulation of mRNA is a crucial step leading to the aberrant expression of cancer-associated genes. It is well known that after the transcription of genomic DNA into pre-mRNA, it needs to undergo 5′ end-capping, splicing, and 3′ end-processing for maturation (Tudek et al., 2018). 3′ end-processing involves recognition of polyadenylation signaling sites (PAS), cleavage, and addition of polyadenylation tails. Approximately 70% of human genes contain multiple PAS (Tian and Manley, 2017). If different PASs are selected during the processing of the 3ʹ end of the mRNA, this will result in the production of multiple transcripts with different coding regions and 3′ untranslated regions (3′ UTRs) for a single gene (Tian and Manley, 2017). This post-transcriptional regulatory mechanism, called alternative polyadenylation (APA), increases the complexity and diversity of the transcriptome and proteome.

With the development of sequencing technology and genomic analysis methods, the mechanism of APA’s role in cancer has received extensive attention (Yuan et al., 2021). Recent studies have shown that APA regulation and the different transcriptional isoforms generated by it are involved in various aspects of tumorigenesis and development. In addition, aberrant expression of factors regulating APA leads to disruption of the APA process, which in turn affects cancer progression. Breast cancer, as one of the cancers threatening women’s health, is closely related to abnormal APA regulation. Therefore, elucidating the biological mechanisms of APA and its regulatory factors in breast cancer will help to comprehensively understand the pathogenesis of breast cancer and provide new ideas for its prevention and treatment.

## 2 The APA process

**FIGURE 1:**
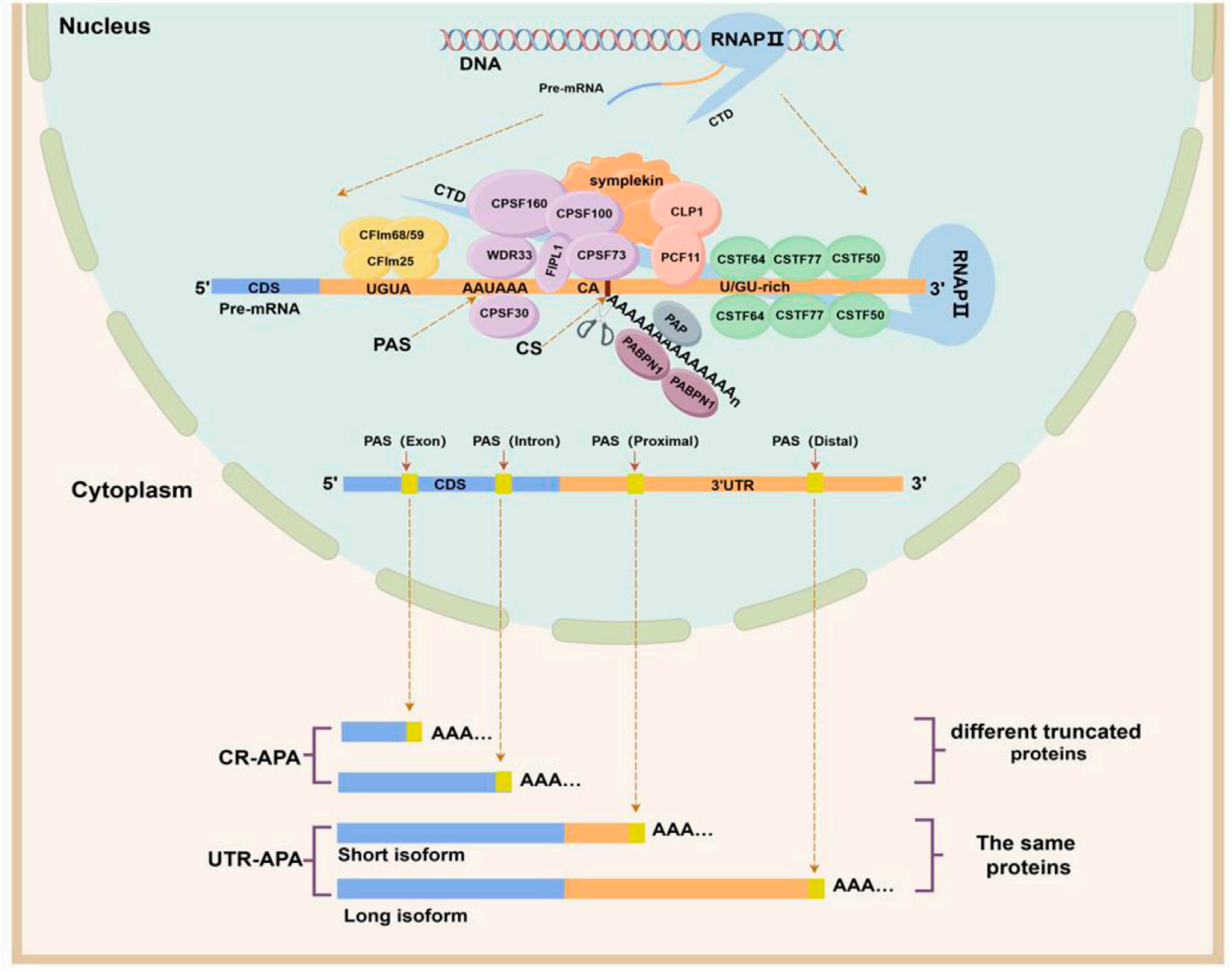
APA formation process and classification. The upper half of this figure shows the cis-acting regulatory elements and trans-acting factors involved in APA formation. The CPSF complex consists of six protein subunits: CPSF160 (CPSF1), CPSF100 (CPSF2), CPSF73 (CPSF3), CPSF30 (CPSF4), FIP1, and WDR33. The CSTF complex consists of three protein subunits: CSTF50 (CSTF1), CSTF64 (CSTF2), and CSTF77 (CSTF3). The CFI complex includes two protein subunits, CFIm25, CFIm59, and CFIm68, and the CFII complex includes two protein subunits, PCF11 and CLP1. The lower half of this figure shows the categories of APAs. Depending on the location of the PAS, alternative polyadenylation can be classified into two categories, namely, untranslated region APA (UTR-APA) and coding region APA (CR-APA). (By Figdraw).

APA is an integrated process accomplished by the synergistic action of multiple components. In this process, a variety of cis-regulatory elements, trans-acting factors, RNA Binding Protein (RBP), and RNA polymerase are required (Figure 1) (Pereira-Castro and Moreira, 2021). Cis-regulatory elements include UGUA sequences, PAS, cleavage site (CS), and U/GU-rich sequences, which are highly conserved. Among them, the PAS is located within a region of 10–30 nucleotides upstream of the CS, and AAUAAA is the classical PAS motif, but other PAS motifs are often used in mammalian cells. In addition, the two nucleotides commonly found at the front of the CS are cytosine and adenine (CA) (Beaudoing et al., 2000). The core trans-acting factors that regulate APA formation are four protein complexes: cleavage and polyadenylation specificity factor (CPSF), cleavage stimulatory factor (CSTF), cleavage factor complexes I (CFIm) and cleavage factor complexes II (CFIIm). In addition, some auxiliary trans factors such as symplekin, poly(A) polymerase (PAP), retinoblastoma binding protein 6 (RBBP6), RNA polymerase II (RNAP II), and nuclear poly(A) binding protein 1 (PABPN1) are involved in APA processing in the form of protein monomers (Pereira-Castro and Moreira, 2021).

These four types of protein complexes are the core processing factors for APA formation and participate in the APA process together by recognizing specific cis-regulatory sequences on pre-mRNA. (I) Assembly stage: CPSF160, a subunit of the CPSF complex, serves as a scaffold to recruit WDR33 and CPSF30 to co-recognize and bind to PAS sequences (Masamha and Wagner, 2018), while the FIPL1 subunit binds to U-rich sequences located between the PAS and the CS. The CSTF64 subunit in the CSTF complex interacts directly with the GU-/U-rich sequence located downstream of the PAS (Beyer et al., 1997). The CFIm25 subunit of the CFIm complex binds specifically to the UGUA motif located upstream of the PAS (Yang et al., 2011), while the PCF11 subunit of the CFIIm complex binds to a G-rich sequence element downstream of the PAS (Pereira-Castro and Moreira, 2021). The carboxy-terminal domain (CTD) of RNAP II and the Symplekin work together as a scaffold to assemble these protein complexes (Zheng and Tian, 2014). (II) Cleavage and tail addition phase: when these complexes are assembled and bound to pre-mRNA, the CPSF73 subunit of the CPSF complex recognizes the CS downstream of the PAS, precisely cleaves the pre-mRNA and adds polyadenylate tails under the action of PAP (Dharmalingam et al., 2022). (III) Termination phase: PABPN1, which binds to the polyadenylated tail sequence, determines when the tail-addition process stops, and after the formation of an adenosine tail of about 50–250 nucleotides, PAP and CPSF73 dissociate and the APA process ends (Ren et al., 2020). In summary, APA processing is so precise that any small change in cis-regulatory elements and trans-acting factors can affect the selection of PAS by pre-mRNAs to determine the length and composition of the 3ʹ end.

## 3 Classification of APA and its biological function in breast cancer

Based on the location of PAS on Pre-mRNAs, APAs can be classified into two main categories (Singh et al., 2018; Pereira-Castro and Moreira, 2021): coding region APA (CR-APA) and untranslated region APA (UTR-APA). For CR-APA, the PAS is located in exons or introns and can alter the coding sequence and 3′UTR of the mRNA to produce truncated protein isoforms with different functions. Notably, if the PAS is located on an exon that does not contain a stop codon, its transcript is rapidly degraded by the uninterrupted decay pathway (Tian and Manley, 2017). UTR-APA, the PAS is located in the 3ʹUTR, resulting in transcription products that contain the same coding frame but have different lengths of the 3ʹUTR (Figure 1). Since a large number of cis-regulatory elements that regulate gene expression exist within the 3ʹUTR, differences in 3ʹUTR length affect the regulatory effects of microRNAs and RBPs on mRNAs, altering mRNA stability, translation efficiency, and protein expression levels.

### 3.1 CR-APA in breast cancer

In breast cancer, the most common CR-APA is intronic polyadenylation (IPA) where the PAS is located in an intron (Table 1). It has been shown that IPA events occur in about 20% of human genes, mostly generating mRNAs of different isoforms encoding different protein products (Tian et al., 2007). Elkon et al. (2012) detected MCF-10A cells in different physiological states by deep sequencing and found that cells in the proliferative state had enhanced cleavage and polyadenylation of intron PAS compared to MCF-10A in the arresting state. Further IPA events of RNF220 and FAM700B genes were detected in cells in the proliferative state by 3′-end RT-PCR analysis (Elkon et al., 2012). IPA is not only seen in normal breast epithelial cells in a proliferative state but also in different types of breast cancer cells or tissues. For example, the ELP5, ERO1A, and PHFT1 genes in MCF7 cells can generate truncated mRNA transcripts through IPA, producing truncated proteins with aberrant functions or dominant negative effects (Turner et al., 2020); PRMT2 Pre-mRNA in breast cancer tissues generates the shorter transcript PRMT2L2 via IPA, which is associated with ERα-positive status and may be involved in breast cancer formation and progression by regulating the estrogen-ERα signaling pathway (Zhong et al., 2011). Confocal microscopy findings showed different intracellular localization of wild-type PRMT2 and PRMT2L2, suggesting that the truncated proteins produced by IPA may alter the cellular localization of the original proteins. In addition, the truncated proteins produced by IPA may also be involved in breast cancer development and progression by inhibiting the function of full-length proteins. In MDA-MB-231 cells, the truncated MAGI3 protein produced by IPA was unable to bind to the oncoprotein YAP compared to the intact MAGI3 protein, and YAP was released, further activating downstream signaling pathways and accelerating the malignant transformation of human breast epithelial cells (Ni and Kuperwasser, 2016).

**TABLE 1:** Different types of APA in breast cancer.

APA types	Breast cancer types	Genes of APA dysregulation	References
CR-APA	Proliferative MCF-10A cell	IPA: RNF220, FAM70B	Ni and Kuperwasser (2016)
	ER + breast cancers	IPA: ELP5, ERO1A, PHFT1, PRMT2	Zhong et al. (2011), Turner et al. (2020)
	TNBC	IPA: MAGI3	Elkon et al. (2012)
UTR-APA	Luminal B breast cancer	3′UTR shorten: DDX5,DVL3, SNRNP200, HEY2, SKI, FOXP4, BHLHE40, U2SURP, CTNNBIP1, PPM1A, CTNNBIP1, SNCA, TIMP3, THRB, PHF12, MXD4, ASB1	Wang et al. (2015)
		3′UTR length: BCL2, PDGFC, TRPS1, STAT6, PIK3R1, COL1A2	
	ER + breast cancers	3′UTR shorten: CDC6, PRELID1, EPS15, NFIA, ADRBK2, SH3GLB1, STX12, ATL3, PPP2R1B, MID2, VKORC1L1, HuR	Akman et al. (2012), Gillen et al. (2017), Tan et al. (2017), Park et al. (2018)
	TNBC	3′UTR shorten: IQCK, USP9X, RPL13, TOP2A, YME1L1, NRAS, Cjun, CCNA2, CCND1, ANAPC5, SNX3	Fu et al. (2011), Akman et al. (2015), Miles et al. (2016)
		3′UTR length: ANAPC13, CDC25B, CDC25C, RAB10, CASP6, DFFA, DFFB, PARP1, DICER1, CDKN2C	
	Unspecified breast cancer	3′UTR shorten: POLR2K, NET1, AGR2, CCND1, IMP1, FGF2, RAB10, SYNCRIP, TMEM237, LRRFIP1, IQCK, TFDP1, SYNCRIP, TNPO1, PMEPA1, FGF2, MITF, Ki67, DICER1, SAP30L, ZHX3, MTURN, C6orf89, APH1B, FAM199X, YIPF6, CRTC1, SEPT6, KLHL36, WDFY3, ZBTB44, ARIH2, PTCH1, KASA	Mayr and Bartel (2009), Lin et al. (2012), Xia et al. (2014), Matoulkova et al. (2017), Xiang et al. (2018), Xue et al. (2018), Yan et al. (2018), Ganapathi Sankaran et al. (2019)
		3′UTR length: CPE135, ATP5S, CCNE1, MAX, SOX2, WT1, NDE1, COL1A2, COL1A1, PPP3CB, ADGRL1, FLT3	

**Note:** The oncogenes in breast cancer are shown in red, the tumor suppressor genes in breast cancer are shown in green, and those with unclear roles in breast cancer are shown in blue.

### 3.2 UTR-APA in breast cancer

UTR-APA is associated with tissue type and cellular status, and its mediated 3′UTR shortening is commonly found in highly proliferative, poorly differentiated cancer cells (Pereira-Castro and Moreira, 2021). 3′UTR shortening has been reported to be widespread in different types of breast cancer (Table 1). Xia et al. (2014) analysed RNA-seq data from seven tumour types, including breast cancer, by DaPars, and identified a large number of highly specific genes associated with tumours, more than 90% of which underwent UTR-APA-mediated 3′UTR shortening. Wang et al. (2015) analysed APA sites in luminal B breast cancer (ER + PR + Her2^+/−^) by sequencing APA sites (SAPAS) method and bioinformatics, and also found a large number of 3′UTR-shortening genes, which are closely related to the spliceosome and Wnt receptor signaling pathway. A large number of 3′UTR-shortening genes have also been found in triple-negative breast cancer (TNBC) tissues, and most of them are associated with tumour cell proliferation and clinical prognosis of patients (Akman et al., 2015; Wang et al., 2019). In addition to the global effect of UTR-APA dysregulation on breast cancer-related genes, it can also affect the proliferation, migration and invasion of breast cancer by regulating the APA patterns of individual oncogenes or tumour suppressor genes. Mayr and Bartel found that the oncogene IGF2BP1/IMP-1 undergoes more 3′UTR shortening in breast cancer tissues compared to paraneoplastic tissues. If the shorter IGF2BP1/IMP-1 mRNA isoforms were transiently transfected into normal breast cell lines, the normal breast cells would undergo malignant transformation and have a significantly higher proliferative capacity (Mayr and Bartel, 2009). PPRELID1 is a gene that promotes tumour cell growth. Gillen et al. (2017) sequenced polyadenylation sites (PAS-seq) and found that the 3′UTR of PRELID1 gene mRNA in ER-positive breast cancer cells was significantly shortened, and its stability and translational efficiency were significantly increased, which in turn increased the growth capacity of tumour cells (Gillen et al., 2017). Ki67, which is closely related to cancer cell proliferation, can also increase its protein expression through 3′UTR shortening, thus enhancing the proliferation ability of breast cancer (Yan et al., 2018). Guo et al. (2022) identified a novel p62 mRNA isoform with a short 3′UTR (p62-SU) associated with breast cancer chemoresistance by RNA sequencing (RNA-seq). Overexpression of P62-SU enhanced the proliferation, migration, invasion and chemoresistance of breast cancer cells compared to the p62 mRNA isoform with full-length 3′UTR (p62-LU) (Guo et al., 2022). In addition, HuR in ER + breast cancer cells (Tan et al., 2017), NRAS and c-JUN in TNBC tissues (Miles et al., 2016) and AGR2 in human breast cancer tissues (Matoulkova et al., 2017) are oncogenes that can evade miRNA-mediated repression through 3′UTR shortening, increase their mRNA stability and translational efficiency, and contribute to the development of breast cancer. Notably, some proliferative signals (e.g., epidermal growth factor and estrogen) can induce further shortening of the 3′UTR of oncogenes (Akman et al., 2012; Akman et al., 2015). The shortening of the 3′UTR caused by UTR-APA not only directly activates proto-oncogenes, but also indirectly downregulates the expression of tumour suppressor genes. This is because UTR-APA produces 3′UTR shortening genes that compete with tumor suppressor genes to form endogenous RNA (ceRNA). Therefore, the originally bound miRNA is released and redirected to tumor suppressor genes with the same miRNA target site, thereby reducing the expression of tumor suppressor genes, such as PHF6 and LARP1 (Park et al., 2018). In addition, 3′UTR shortening can regulate the stability of mRNAs of proto-oncogenes and tumor suppressor genes through RBP and affect their protein expression, thus exerting a pro-carcinogenic effect (Masamha and Wagner, 2018).

In conclusion, CR-APA and UTR-APA are closely related to breast cancer. CR-APA plays a relatively limited role, mainly by changing the protein structure, affecting the function of oncoprotein and tumor suppressor protein, and promoting tumor progression. UTR-APA, which is more widely distributed, participates in the occurrence and development of breast cancer by directly activating proto oncogenes, indirectly inhibiting tumor suppressor genes by interfering with ceRNA networks, and mediating RBP to affect the stability of key genes. It is worth noting that not all genes are regulated by APA, and not all APA mediated 3′UTR shortening can increase protein expression levels. Only when miRNA or RBP targeting the 3′UTR gene is significantly expressed, its stability and expression level will be affected (Masamha and Wagner, 2018).

## 4 Abnormal APA regulators associated with breast cancer

It has been shown that aberrant expression of APA regulatory factors can affect PAS selection and cellular phenotype. Most of the APA regulatory factors are involved in APA processing, and some of them also directly regulate the disease process. In the following, we will discuss some of the factors that have been shown to be involved in the regulation of APA and explore their biological functions in breast cancer (Table 2).

**TABLE 2:** Abnormal APA regulatory factors related to breast cancer.

APA regulators	Role in PAS choice	Expression in BC	Genes affected	References
CFIm25	Promotes distal PAS selection	Downregulation	CPSF6, E-cadherin, Occludin, ZEB-1, Vimentin	Wang et al. (2020a), Tamaddon et al. (2020)
CFIm68	Promotes distal PAS selection	Upregulation	A-to-I RNA Editing	Binothman et al. (2017)
PAPOLA	Promotes proximal PAS selection	Upregulation	CCND1	Li et al. (2017), Komini et al. (2021), Mohanan et al. (2022)
PABPN1	Promotes distal PAS selection	Upregulation	NUDT5, HDAC7, HDAC1, MMS22L, DHX36, MUM1, PHF21A	Ichinose et al. (2014), Xiang et al. (2018), Wang et al. (2020b), Kurozumi and Lupold (2021), Chen et al. (2023)
hnRNPC	Promotes proximal PAS selection of 3′UTR and inhibits the use of intron PAS	Upregulation	dsRNA	Gruber et al. (2016), Wu et al. (2018), Lv et al. (2021), Dharmalingam et al. (2022), Mo et al. (2022)
U1 snRNP	Suppress premature cleavage and polyadenylation within introns	Upregulation	TNFAIP2, E2F2, CDK4	Kaida et al. (2010), Berg et al. (2012), Kurozumi and Lupold (2021), Lu et al. (2023)
CPEB1	Promotes proximal PAS selection	Downregulation	MMP9	Bava et al. (2013), Nagaoka et al. (2016), Pereira-Castro and Moreira (2021), Sovijit et al. (2021)
CSTF2	Promotes proximal PAS selection	Upregulation	HuR	Xia et al. (2014)
RBBP6	Promotes proximal PAS selection	Upregulation	c-Fos, c-Jun, p53, bcl2, bax, bak1, bad, caspase8	Di Giammartino et al. (2014), Motadi et al. (2018), Mbita et al. (2019)
CPSF1	Unknown	Upregulation	PIK3C2G, IL21A, RAD51D, MMS22L, SPIDR, MTA3, MIA3	Wang et al. (2020b), Guo et al. (2022)

### 4.1 CFIm25 and CFIm68

CFIm25 (also known as CPSF5, or NUDT21) and CFIm68 (also known as CPSF6) are both subunits of the CFIm complex. It has been demonstrated that both CFIm25 and CFIm68 are key regulators of APA, involved in PAS selection and regulation of 3′ UTR length (Jafari Najaf Abadi et al., 2019; Kurozumi and Lupold, 2021). Knockdown of both regulators promotes preferential selection of the proximal PAS, resulting in an overall shortening of the 3ʹUTR of the transcript, which increases the stability and expression level of the target gene. In addition, CFIm25 influences the selection of PAS by chromatin-regulated genes and is involved in cell fate reprogramming (Brumbaugh et al., 2018). Although CFIm25 and CFIm68 act in concert in PAS selection, their roles are completely opposite in breast cancer. IHC analysis shows that CFIm25 is under-expressed in breast cancer tissues compared to tissues with benign breast disease. Overexpression of CFIm25 inhibits cancer cell proliferation, migration, invasion and Epithelial-mesenchymal transition (EMT) (Wang et al., 2020a). In addition, CFIm25 itself can be regulated by miRNAs as a target gene, suggesting that regulating the expression level of CFIm25 by miRNAs is expected to improve cancer treatment (Tamaddon et al., 2020). CFIm68, on the other hand, is involved in breast cancer progression as an oncogenic factor (Binothman et al., 2017). Binothman et al. (2017) found that high expression of CFIm68 in BC was associated with poor patient prognosis. Knockdown of CFIm68 significantly reduced cell viability, inhibited colony forming ability and induced apoptosis in invasive breast cancer cells. The specific molecular mechanism is that CFIm68 promotes tumourigenesis by enhancing the A-to-I RNA editing process, an RNA processing mechanism that is one of the major factors driving tumourigenesis (Binothman et al., 2017). Interestingly, CFIm25 was able to negatively regulate CFIm68, and knockdown of CFIm68 reversed cancer cell migration and invasion induced by low CFIm25 expression, but the mechanism by which CFIm25 and CFIm68 interacted has not been clarified (Wang et al., 2020a).

### 4.2 PAPOLA

The poly (A) polymerase (PAP) family is a core member of the polyadenylation mechanism, and PAP is mainly responsible for the addition of the poly (A) tail of mRNA (Mohanan et al., 2022). It is known that there are three subtypes of typical PAPs, encoded by different genes, namely, PAPOLA, PAPOLB, and PAPOLG. Through RNA sequencing and bioinformatics analysis, Li et al. (2017) found that the 3ʹUTR of the gene was prolonged after PAPOLA silencing, indicating that PAPOLA has the ability to promote proximal PAS selection. PAPOLA is generally overexpressed in breast cancer, and overexpression of PAPOLA can shorten the 3ʹUTR of cyclin D1 (CCND1) mRNA, increase the protein expression of CCND1, and then promote the proliferation of breast cancer cells (Komini et al., 2021).

### 4.3 PABPN1

PABPN1 is a key factor involved in APA formation, which not only increases the ability of PAP to sustain tail addition, but also controls the length of the poly(A) tail (Kurozumi and Lupold, 2021). PABPN1 was identified as a potent repressor of proximal PAS selection, and its reduced expression leads to an overall shortening of the 3′UTR (Xiang et al., 2018). PABPN1 has been reported to be associated with human cancer progression. PABPN1 is lowly expressed in non-small cell lung and bladder cancers and functions as a tumor suppressor (Ichinose et al., 2014; Chen et al., 2023). However, in TNBC, PABPN1 is highly expressed, and knockdown of PABPN1 inhibits cell proliferation, promotes apoptosis, leads to cell cycle redistribution, and reverses APA events in genes associated with tumourigenesis, proliferation, metastasis, and chemo-sensitivity in breast cancer (Wang et al., 2020b).

### 4.4 hnRNPC

Heterogeneous nuclear ribonucleoprotein C (hnRNPC) is an RNA-binding protein involved in the regulation of APA (Dharmalingam et al., 2022). Gruber et al. (2016) sequenced the 3ʹ end of the pre-mRNA after knockdown of hnRNPC and found that knockdown of hnRNPC increased the use of intronic PAS and an increase in the occurrence of IPA, which resulted in the production of truncated proteins or the effective downregulation of functional full-length transcripts production. Knockdown of hnRNPC also increases the use of distal U-rich PAS in the 3′UTR, leading to overall lengthening of the 3ʹUTR of target genes (Mo et al., 2022). It is suggested that aberrant expression of hnRNPC can dysregulate the IPA and UTR-APA processes of target genes, which are involved in tumour development. MTHFD1L is a key enzyme in mitochondrial folate metabolism, which is closely related to cell proliferation (Fischl et al., 2019). In metastatic colonic epithelial cells, overexpression of hnRNPC can both upregulate the content of functional full-length transcript isoforms of MTHFD1L through IPA, and shorten the 3ʹUTR of MTHFD1L to improve translational efficiency, which together increase the protein expression level of the full-length MTHFD1L and play an oncogenic role. In breast cancer, high expression of hnRNPC inhibits the interferon response and promotes breast cancer cell proliferation by down-regulating dsRNAs, and high hnRNPC expression is associated with advanced clinical stage and shorter survival time (Wu et al., 2018; Lv et al., 2021). These regulated dsRNAs are mainly derived from pre-mRNA introns carrying hnRNPC binding sites, suggesting that the oncogenic role of hnRNPC in breast cancer may be associated with increased IPA occurrence (Wu et al., 2018).

### 4.5 U1 snRNP

U1 snRNP is involved in splice site recognition and protects nascent RNAs from premature cleavage and polyadenylation (PCPA) through a mechanism known as “Telescripting” thus ensuring transcriptome integrity and participating in the regulation of mRNA length (Kaida et al., 2010; Berg et al., 2012; Kurozumi and Lupold, 2021). When U1 snRNP is inhibited, many cryptic polyadenylation signals (PASs) located on the pre-mRNA intron or at other locations are activated, leading to PCPA. Therefore, inhibition of U1 snRNP enhances the usage of proximal PASs, leading to an overall shortening of transcripts. Lu et al. (2023) found that the subunit of U1 snRNP, SNRPC, is beneficial for the RNAP II-controlled transcriptional process. In triple-negative breast cancer, SNRPC enhances cancer cell invasiveness by accelerating RNAP II-controlled oncogene transcription (Lu et al., 2023).

### 4.6 CPEB1

Cytoplasmic polyadenylation element binding protein 1 (CPEB1) can both control poly(A) tail length to regulate mRNA translation and influence the overall 3′UTR length (Bava et al., 2013). CPEB1 mediates the shortening of the mRNA 3′UTR in the nucleus, mainly by promoting the use of proximal PAS by CPSF (Pereira-Castro and Moreira, 2021). Nagaoka et al. (2016) found that CPEB1 was lowly expressed in highly metastatic breast cancer cells, whereas the metastasis-promoting factor matrix metalloproteinase 9 (MMP9) was highly expressed. Experimental analyses revealed that CPEB1 deletion leads to lengthening of the poly (A) tail of MMP9 mRNA and enhances translation efficiency (Nagaoka et al., 2016). In addition, CPEB1 is regulated by estrogen, which promotes the proliferation and migration of breast cancer cells by down-regulating CPEB1 expression (Sovijit et al., 2021).

### 4.7 Others

Many APA regulators are abnormally expressed in breast cancer. They are involved in breast cancer development by regulating the selection of oncogenes and tumor suppressor genes PAS, affecting their mRNA stability and translation efficiency. Among them, CFIm25, CFIm68, and PABPN1 promote the selection of distal PAS, and their overexpression can lead to global shortening of 3′UTR. CPSF1, CSTF2, CSTF3, hnRNPC, PCF11, CPEB1, RBBP6, and PAPOLA promote the use of proximal PAS, and their overexpression can lead to global elongation of 3′UTR (Table 2).

## 5 APA in the diagnosis and prognosis of breast cancer

In breast cancer, UTR-APA or CR-APA dysregulation occurs in many important genes, resulting in differences in mRNA 3′UTR length, stability, translational efficiency, as well as its protein expression and function, which together constitute the breast cancer-specific APA signature, which can be used in combination or individually as a biomarker for the diagnosis and prognosis of breast cancer. Zhang et al. (2020) collated and analyzed the APA events and clinical information of BC patients in the TCGA database by LASSO regression and multivariate Cox regression, and classified BC patients into high-risk and low-risk groups based on the median risk score. Kaplan-Meier survival analysis and receiver operating characteristic curve (ROC) analysis showed that overall survival (OS) and Recurrence-free survival (RFS) were better in the low-risk group than in the high-risk group, and the APA characteristics have good predictive ability for survival and recurrence in BC patients (Zhang et al., 2020). Kim et al. (2019) analyzed the APA patterns and gene expression levels of 515 scRNA-seq datasets from 11 breast cancer patients and found that there were differences in APA patterns in gene sets between tumor and non-tumor cells. In addition, they analyzed scRNA-seq data from 3 patients with glioblastoma and 1 patient with renal cell carcinoma and found that different types of tumor cells could be distinguished based on unique patterns of gene expression and 3′UTR length changes in the APA gene set. They also demonstrated that immune-specific APA features in breast cancer can be used as prognostic markers for early breast cancer (Kim et al., 2019). TNBC is an aggressive malignant tumor with a high degree of heterogeneity. Sentinel lymph node biopsy is the standard method for surgical staging of clinically axillary-negative operable TNBC. However, predicting whether TNBC has lymph node metastasis by tissue biopsy is both damaging to the patient and unreliable. Therefore, Wang et al. (2019) developed and constructed a model based on 3′UTR length and tumor size that can be used to identify TNBC patients at low risk of lymph node involvement, allowing them to be spared axillary surgery. However, the model requires a prospective study to validate it. In addition, Gillen et al. (2017) found that APA events of PRELID1 mRNA are important predictors of clinical prognosis in patients with breast cancer subtypes. In conclusion, the overall characteristics of APA or the APA patterns of important genes are tumor-specific and may serve as new targets for breast cancer diagnosis and prognosis.

## 6 Conclusion and perspective

APA, as a ubiquitous gene expression regulatory mechanism in eukaryotes, is associated with cell proliferation rate and differentiation status. Breast cancer is a highly proliferative and poorly differentiated disease, and a large number of studies have shown a close relationship between APA and breast cancer. APA dysregulation can promote the development and progression of breast cancer by increasing the expression of proto-oncogenes and decreasing the expression of oncogenes, and the aberrant expression of APA-associated regulators can also lead to the disruption of the APA process in target genes, which can affect the biological function of breast cancer. Moreover, APA is tumor-specific, suggesting that it could be a new target for breast cancer diagnosis and treatment. In the future, the APA pattern of disease-causing genes can be corrected by genome editing, RNA editing, antisense oligonucleotide, or small molecule targeting to restore their gene expression and function, so as to achieve therapeutic effects. In summary, clarifying the regulatory mechanism of APA is of great significance to the prevention and treatment of breast cancer, and future research can focus on the following three aspects. (I) In-depth investigation of the sequential or causal relationship between APA and the process of cellular carcinogenesis; (II) Continue to identify specific APA events as well as APA regulatory factors and perform functional validation; (III) Focus on the development of computational tools and databases that can recognize CR-APA. Ultimately, as APA research continues, it will enhance the diagnostic techniques and treatment methods of breast cancer, elucidate its pathogenesis, improve the survival rate and quality of life of cancer patients, and at the same time lead to medical advances in tumor treatment.
